# The Impact of Peer Support on Patient Outcomes in Adults With Physical Health Conditions: A Scoping Review

**DOI:** 10.7759/cureus.17442

**Published:** 2021-08-25

**Authors:** Ellie Grant, Louise Johnson, Apostolos Prodromidis, Peter V Giannoudis

**Affiliations:** 1 Clinical and Health Psychology, Leeds Teaching Hospitals NHS Trust, Leeds, GBR; 2 Academic Department of Trauma and Orthopaedics, University of Leeds, Leeds, GBR

**Keywords:** peer support, patient outcome research, patient-centered outcomes research, rehabilitation psychology, rehabilitation program

## Abstract

Little is known about the impact of peer support programmes on physical health populations or on the methods used to evaluate such programmes. The present study undertakes a scoping review of research related to peer support programmes or interventions in physical health populations, guided by the Preferred Reporting Items for Systematic Reviews and Meta-Analyses extension for scoping reviews (PRISMA-ScR).

The search was carried out across the Medline, PsycINFO, and Cochrane databases and focused on papers that evaluated peer support intervention(s) in adults with physical health conditions. The search identified an initial 7,903 records, which were narrowed down to 21 records that met the inclusion criteria; their findings were narratively synthesized.

The scoping review found considerable heterogeneity among eligible records in terms of their study design, outcome measurements and findings reported. Qualitative methods of evaluation generated more consistent findings compared to objective outcome measures and suggested that peer support was beneficial for patients’ health and wellbeing by reducing feelings of isolation and creating a sense of community as well as providing an opportunity for information consolidation. The scoping review highlights the inconsistencies in methods used to evaluate peer support interventions and programmes in healthcare settings among different physical health populations. It also draws attention to the lack of peer support research in particular areas, including in acute physical health populations such as in major trauma. The scoping review emphasizes the need for future studies to address this gap in peer support research.

## Introduction and background

‘Peer support’ is a process that involves individuals drawing on lived experience or shared characteristics to provide knowledge, experience, emotional assistance, practical help, and social interaction to help each other [1]. Peer support can take many forms such as one-to-one, group work, and online and telephone support sessions. It can be informal or more formalised, with trained peer supporters. Peer support in healthcare settings can be standardized by content or function. A global initiative in 2010 that aimed to promote best practices in peer support for health around the world adopted a functional approach to the standardization of peer support and identified the following four core functions: (1) to provide practical support; (2) to provide social and emotional support; (3) to ensure linkages to clinical care and community resources and (4) to provide ongoing support [2].

There is an abundance of peer support research in healthcare that focuses on mental health and HIV, and now more attention is being directed towards alternative population groups, including those with long-term physical health conditions. Research has also shown that peer support is especially valued by young people and British Black, Asian and Minority Ethnic (BAME) adults [3]. Those from “hardly reached” populations have also been found to benefit from peer support interventions, suggesting that peer support is a broad and robust strategy for reaching these groups that health services often fail to engage [4].

There is an increasing evidence base suggesting a range of benefits of peer support for people across various populations. A review commissioned in 2015 that included more than 1,000 research studies concluded that peer support has the potential to improve experience, psycho-social outcomes, behaviour, health outcomes and service use among people with long-term physical and mental health conditions [5]. A further review of the literature showed that peer support leads to significant improvements for people with long-term physical and mental health conditions across various outcomes such as quality of life, social functioning and perceived support, individual’s knowledge, skills and confidence to manage their health and care and physical functioning and ability to self-care [6].

The objectives of this scoping review were twofold. First, to gain a better understanding of how various peer support programmes impact patient outcomes in patients with physical health conditions. Second, to gain an insight into how peer support programmes are evaluated. This method of review was selected to enable outcomes to be synthesized in order to provide more context to the evidence base and inform clinical practice. The following research question was generated: What is known about peer support programmes in physical health populations, and how are these programmes evaluated in terms of their effectiveness?

## Review

Protocol and registration

The final protocol was registered prospectively with Figshare (https://figshare.com/articles/preprint/Scoping_review_protocol_The_impact_of_peer_support_on_patient_outcomes_in_adults_with_physical_health_conditions/15178059) and the review was conducted with reference to this protocol.

Eligibility criteria

Papers were eligible if they: (1) evaluated a peer support programme in a physical health setting(s) including in primary, secondary or community care settings; (2) included individuals with any physical health condition; (3) included any type of peer support programme/intervention; (5) were published in peer-reviewed journals.

Information sources

The search was carried out across multiple healthcare databases: Medline (Interface: EBSCOhost), PsycINFO (Interface: Healthcare Databases Advanced Search) and the Central (Interface: Cochrane Library). Search results were imported into a citation manager software (Endnote), and duplicates were removed via a combination of the removal of duplicates function on the programme and a manual check by one of the authors.

Search

The following search terms were used to search for eligible studies in all databases up to July 01 2021. Search terms were intentionally few to reduce the likelihood of omitting papers that may not have specifically indicated including participants from within the broad umbrella of those with physical health conditions but rather mentioned the condition itself. Search terms were limited to within titles and abstracts of studies.

1. Peer support*

AND

2. Evaluation* OR Review*

Selection of sources of evidence

Screening of papers was guided by the Preferred Reporting Items for Systematic Reviews and Meta-Analyses (PRISMA) framework. One reviewer screened all of the publications, including the title, abstract and full-text screening and was supported by the other reviewers. All reviewers were involved in determining the quality of the screening process and any queries or disagreements were resolved through in-depth discussion.

Data charting process

Data from selected studies were extracted using a standardized data collection form amended for this review. This tool captured information related to the characteristics of studies, including study aim(s), design, population demographics, nature of intervention(s), description of outcome(s) and method(s) of evaluation of data. One reviewer extracted data from the studies with guidance from the other two reviewers. Data were then added to the characteristics of sources of evidence table (Appendix).

Data items

Data abstracted included country of origin, population group, type of peer support programme or intervention, including method of delivery, evaluation methods used, main outcomes, including objective measures for quantitative studies, and derived themes for qualitative studies and conclusions.

Synthesis of results

Studies were grouped by their method of evaluating the peer support programme (either quantitatively or qualitatively). Synthesis of quantitative findings within studies involved summarizing the population group, peer support intervention(s) (including mode of delivery and evaluation methods) and primary outcome measures described. Similar qualitative findings between studies were grouped and over-arching themes are discussed.

Results

Selection of Sources of Evidence

**Figure 1:**
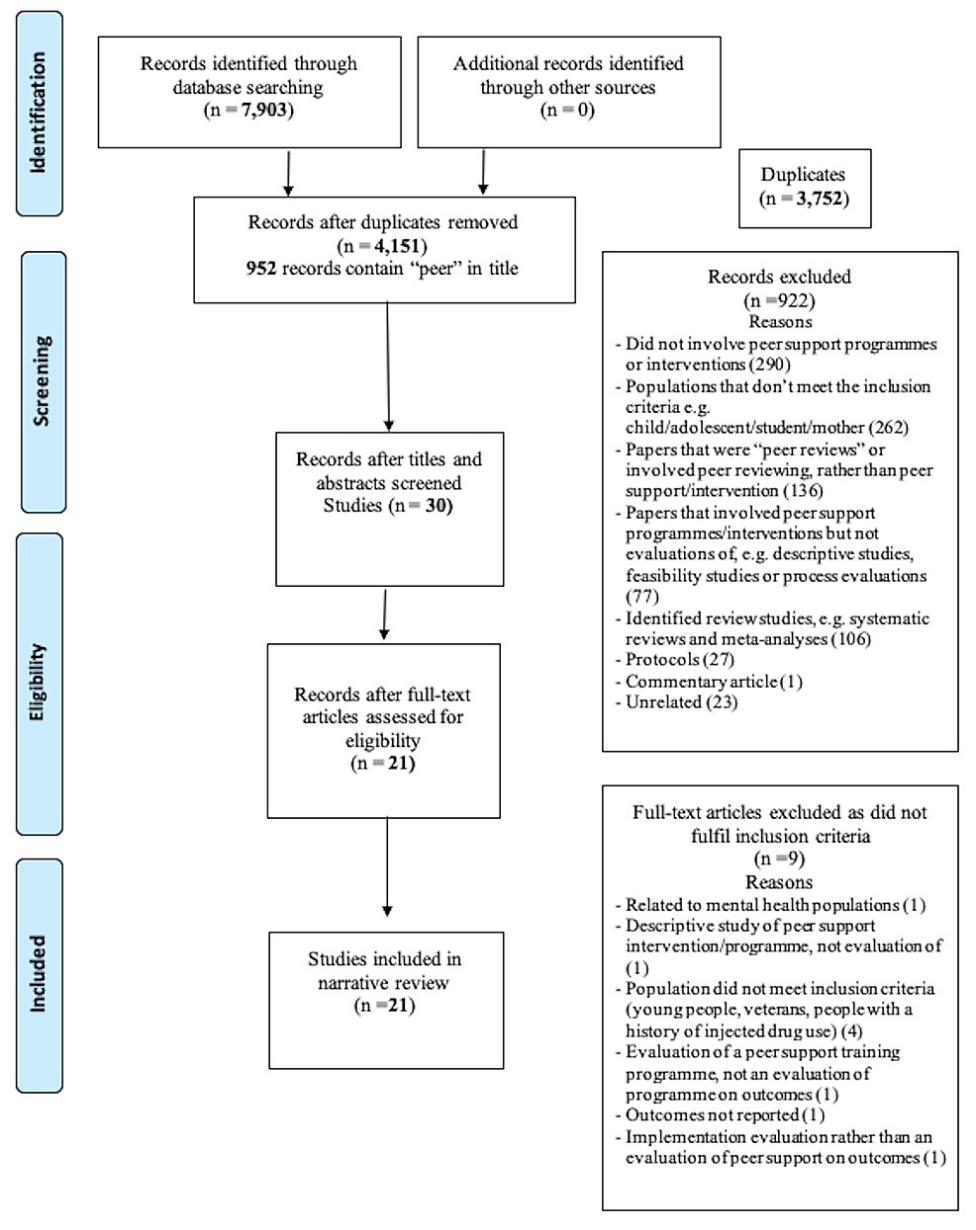
PRISMA diagram of included studies PRISMA: Preferred Reporting Items for Systematic Reviews and Meta-Analyses

Following the removal of duplicates, a total of 4,151 studies remained. Figure 1 shows the PRISMA flow diagram used for the identification of eligible studies [7].

Characteristics of Sources of Evidence

Sample sizes of the included studies varied considerably, ranging from eight participants [8] to 1,229 participants [9]. There was considerable heterogeneity in the population groups of included studies. Seven studies included patients with type 2 diabetes (33.3%) [9-15], five studies included cancer populations (24%) [8,16-19], two studies included patients with spinal cord injuries (9.5%) [20-21], seven studies (33.3%) included participants from other physical health populations; stroke [22], fibromyalgia [23], alopecia [24], polycystic ovary syndrome [25], HIV [26], cardiac [27] and burn injury patients [28].

Peer support interventions

Mode of Delivery

Table 1 shows variation in studies for the mode of delivery of peer support.

**Table 1:** Mode of delivery of peer support.

Author(s) [ID]	% of studies	Mode of delivery of peer support
Smith, Paul, Kelly et al., 2011 [11]; Shen, Wang and Edwards, 2017 [13]; Ono, Tsuyumu, Ota et al., 2017 [17]; Legg, Occhipinti, Ferguson et al., 2011 [18]	19%	Facilitated programme of face-to-face peer support led by trained peer supporters
Power and Hegarty, 2010 [8]; Piatt, Rodgers, Xue et al., 2018 [15], Clark, Munday and McLaughlin et al., 2012 [27]	14%	Facilitated programme of face-to-face peer support led by both peer supporters and health professionals
Percy, Gibbs, Potter et al., 2009 [25]	5%	Facilitated programme of peer support led by health professionals alone
Chan, Sui, Oldenburg et al., 2014 [10]; Dale, Caramlau, Sturt et al., 2009 [14], St-Pierre, Bouchard, Gauthier et al., 2018 [16]; Gotay, Moinpour, Unger et al., 2007 [19]	19%	Telephone-based peer support programme with trained peer supporters
Muller, Toth-Cohen and Mulcahey, 2014 [22]	5%	Broad programme of support with peer support components
Wingate, Graffy, Holman et al., 2017 [9]; Ayala, Ibarra, Cherrington et al., 2015 [12]	10%	Mixed delivery of peer support (telephone and face-to-face)
Haas, Price and Freeman, 2013 [20]; O’Dell, Earle, Rixon et al., 2019 [21]; Sallinen, Kukkurainen and Peltokallio et al., 2011 [23]; Iliffe and Thompson, 2019 [24]; Monroe, Nakigozi, Ddaaki et al., 2017 [26]; Grieve, Shapiro, Wibbenmeyer et al., 2020 [28]	29%	No peer support intervention but evaluations of previously completed peer support programmes

Evaluation Methods

Nine studies (43%) utilized quantitative means of outcome evaluation in order to determine the efficacy of peer support programmes or interventions [9-11,15,18-19,27-29]. Nine studies (43%) involved exploratory qualitative analyses of participant perceptions, feedback or experiences of peer support programmes [8,16-17,20-21,23-26]. Three studies (14%) incorporated both quantitative and qualitative analyses to evaluate peer support [13-14,22].

Primary Outcomes

Self-report measures used to assess primary outcomes are presented in Table 2.

**Table 2:** Measures used to assess primary outcomes. *No description of measure used to assess wellbeing in the paper

Author(s), [ID]	Outcome assessed	Scale/measure used
Psychological		
Smith, Paul, Kelly et al., 2011 [11]	Wellbeing	Study does not report on the measure used*
Legg, Occhipinti, Ferguson et al., 2011 [18]	Psychological affect	The Hospital and Anxiety Depression Scale (HADS)
Shen, Wang and Edwards, 2017 [13]	Self-efficacy	The Chinese Diabetes Self-Efficacy Scale (C-DSES)
Dale, Caramlau, Sturt et al., 2009 [14]	Self-efficacy	The Diabetes Management Self-Efficacy Scale (DMSES)
Shen, Wang and Edwards, 2017 [13]	Quality of life	The Medical Outcome Study 36-item Short-Form Health Survey (SF-36)
Rodgers, Xue et al., 2011 [15]	Diabetes distress	Diabetes Distress Scale (DDS)
Gotay, Moinpour, Unger et al., 2007 [19]	Psychological distress	Cancer Rehabilitation Evaluation System-Short-Form (CARES-SF)
Gotay, Moinpour, Unger et al., 2007 [19]	Depressive symptoms	Center for Epidemiologic Studies Depression Scale (CES-D)
Functional/behavioural/social		
Muller, Toth-Cohen and Mulcahey., 2014 [22]	Healthy adjustment after stroke	The Stroke Impact Scale (SIS)
Muller, Toth-Cohen and Mulcahey., 2014 [22]	Home integration, social interaction and productivity	The Community Integration Questionnaire (CIQ)
Grieve, Shapiro, Wibbenmeyer et al., 2020 [28]	Social participation	The Life Impact Burn Recovery Evaluation Profile
Clark, Munday and McLaughlin et al., 2012 [27]	Physical activity level	7-day Physical Activity Recall Questionnaire (and pedometers)
Clark, Munday and McLaughlin et al., 2012 [27]	Support for physical activity	The Social Support in Exercise Survey
Shen, Wang and Edwards, 2017 [13]	Social support	The Medical Outcome Study Social Support Survey (MOS-SSS)
Shen, Wang and Edwards, 2017 [13]	Self-management behaviours	The Chinese Diabetes Self-care Scale (C-DSCS)
Rodgers, Xue et al., 2011 [15]	Self-management behaviours	Self-monitoring of blood glucose

Psychological Outcomes

The psychological outcome measures cited across included studies varied greatly, with self-efficacy the most commonly reported psychological outcome. A community-based peer-led diabetic self-management programme that involved face-to-face peer support, concluded that self-efficacy significantly improved during the 12-week study period [13]. Notably, the intervention itself centred around self-efficacy enhancing group activities, therefore the programme was tailored to impact this particular outcome. Quality of life among participants, however, did not change significantly. A further study that evaluated a telephone peer-delivered intervention for individuals with type 2 diabetes, found no statistically significant difference in self-efficacy scores at six months [14]. However, multiple differences existed between the studies, despite similarities in the population group and the targeted outcome construct. These differences included the tools used to assess self-efficacy (see Table 2), the delivery of peer support itself (group versus telephone) and the length of time at follow-up (4 versus 6 months).

One study reported a beneficial impact of peer support on diabetes-related distress [15]. In this study, participants were individuals with type 2 diabetes and were randomly allocated to either the intervention group, which involved diabetes self-management education alongside peer-led diabetes self-management support or the Enhanced Usual Care (EUC) group, which was similar to the intervention group, however, it had no components of peer support. The study revealed that peer-led diabetes self-management support was more effective than EUC at improving diabetes distress [15].

One study reported no impact of peer support on psychological outcomes. This study considered the impact of a peer-delivered telephone intervention for women experiencing a breast cancer recurrence [19]. The study concluded that at the three-month follow-up, there were no differences in psychological distress or depressive symptoms between the intervention and control groups.

One study detected a possible detrimental impact of peer support on psychological outcomes for individuals with type 2 diabetes. This randomised controlled trial (RCT) involved a peer-led face-to-face peer support programme that took place over a two-year period. At the two-year follow-up, it was concluded that for the intervention group, there was a reduction in wellbeing (non-significant) compared to the control group [11]. Importantly, the measure used to assess wellbeing was not reported in this paper, thereby it is difficult to assess its validity and reliability.

Two studies [10,18] reported that peer support was more effective in psychological outcomes in population groups that experienced higher levels of negative psychological affects compared to those with low levels. The first study involved telephone-based peer-support and reported that participants with greater negative emotions seemed to benefit significantly more in terms of their psychological health compared with patients with lower levels of negative emotions. These participants also showed greater treatment compliance and reduced hospital admissions [10]. The second study included a peer-delivered face-to-face peer support programme for individuals with breast cancer and concluded that participants that engaged in positive upward comparison to the breast cancer survivor and who also regarded their cancer diagnosis as more threatening in the first instance, appeared to benefit psychologically by showing lower levels of depressive symptoms [18]. These findings suggest that peer support interventions could be more effective in individuals that demonstrate high levels of negative psychological affect.

Functional, Behavioural and Social Outcomes

The impact of peer support on reported functional, behavioural and social outcomes is mixed. A study that evaluated long-term social reintegration outcomes for burn survivors concluded that those who reported peer support attendance had better social interaction scores than those who did not. This included fewer restrictions in participating in social activities, relating and maintaining friendships and dealing with strangers [28]. A study that evaluated a community-based peer-led diabetic self-management programme concluded that social support and self-management behaviours improved significantly during the 12-week study period [13]. Another study revealed that peer-led diabetes self-management support was as effective as Enhanced Usual Care in helping participants to maintain self-monitoring of their blood glucose levels [15].

In comparison, a longitudinal study that involved a facilitator-led programme of face-to-face peer support alongside group-based education for people with heart disease who had completed centre-based cardiac rehabilitation reported that social support did not change between baseline and 12-month follow-up [27]. Additionally, there were no changes in the amount of physical activity reported among programme participants.

Physical Health Outcomes

One study in this review reported a positive impact of peer support on physical health outcomes in individuals with uncontrolled diabetes [12]. A mixed peer-delivered peer support programme concluded that peer support was effective at reducing glycated haemoglobin in intervention versus usual care arm participants [12].

In contrast, an RCT that evaluated the effect of a telephone-based peer support programme in patients with type 2 diabetes found that peer support did not improve cardiometabolic wellbeing [10]. Another study involving participants with type 2 diabetes that utilized a programme of peer-led face-to-face peer support concluded that peer support did not significantly improve physical outcomes (haemoglobin levels, systolic blood pressure and cholesterol levels) [11].

Economic Outcomes

An economic evaluation of a mixed delivery peer support intervention concluded that peer support was associated with lower overall total healthcare costs, which were largely due to a decrease in hospitalization expenses [9]. The evaluation also found that the intervention was associated with a modest increase in out-of-pocket costs for participants and implementation costs. The authors suggested that the increase in out-of-pocket costs may be explained by an increase in participants’ engagement in self-care activities. This study was the only one included in the review that considered economic outcomes following peer support therefore there is a limited evidence base to validate its findings. Moreover, this study reported findings over a relatively short time period (8-12 months).

Qualitative Findings

Some participants described peer support as “a significant turning point in their lives” [23] and something that had a “major personal impact” [25]. Some participants reported that peer support allowed them to “dare to be oneself” [23] whilst others expressed their view that peer support is essential to patients who undergo rehabilitation in a general hospital [20]. A common theme reported among the included studies was peer support leading to a sense of belonging or feeling as though participants were part of a community, which led to reduced isolation and feeling understood [13,16-17,23]. Another key theme was peer support as a means of providing and/or consolidating information [8,22,26]. Peer support proved helpful for some participants in their decision-making [8,16]; others described peer support as responsible for feelings of empowerment [23,25] as well as helpful in aiding self-management behaviours [25].

Much of the qualitative findings suggest that peer support can be beneficial for individuals across various population groups. However, some limitations of peer support were discussed among these positive findings. For example, one study reported that participants experienced raised anxiety related to the future, occasional hopelessness and despair as a result of seeing others with more severe functional disabilities [23]. Furthermore, participants with spinal cord injuries and healthcare practitioners reported in a study that the timing of peer support is essential, as it is not always feasible to deliver in the acute stages of injury [21]. Some practical limitations of peer support programmes were offered, notably matching limitations, strict management of personal information [17] and participants wanting more time to engage in peer support [8].

Discussion

The heterogeneity in findings within the included studies is clear across the various outcomes, namely, psychological, functional/social/behavioural, health and economic. Studies that included qualitative analysis as their method of evaluating peer support programmes appear to offer more consistency in terms of findings, with most suggesting a beneficial impact for participants. This could mean that the impact of peer support is experienced by participants in various settings, however, the actual impact may not be observable or measurable.

There was considerable variation in the methods used for the delivery of peer support in the included studies. It is therefore difficult to review study findings as a collective. Some interventions focused predominantly on the delivery of peer support as its main component, whilst other interventions focused heavily on education methods with additional peer support. Consequently, it would not be appropriate to attribute peer support as the main influencer of outcomes.

Noteworthy, the majority of included studies did not report any long-term outcomes of associated peer support interventions. One study that did, discussed negative feelings reported by participants as a result of seeing others with more severe functional disabilities [23]. This suggests that the functional capabilities of peer supporters as well as the level of exposure to other peers with more severe disabilities need to be considered when developing a peer support programme. Future studies aiming to evaluate peer support programmes should seek to include longitudinal follow-up outcome measures.

Studies that included a measurable peer support component ranged considerably in terms of the length of time of the delivery of peer support, from four weeks [19] to two point eight (2.8) years [16]. Attendance in peer support programmes/interventions was episodic and the number of peer support sessions within the study duration differed enormously. For example, one study [22] reported a sample size of 13, however, only three (23%) participants attended all of the described peer support sessions.

There was considerable variation in the measures used to quantify the impact of peer support. For example, the most commonly cited psychological outcome measure was self-efficacy with two of the included studies reporting this outcome [13-14]. However, the methods used to assess this psychological construct were different for each study (Table 1). Moreover, the term ‘self-efficacy’ was used in these studies to reflect individuals’ experience with their diabetes, rather than self-efficacy as a general psychological construct. Further condition-specific outcomes reported in the included studies were healthy adjustment after stroke [22] and diabetes distress [15]. One of the aims of this review was to draw conclusions on the impact of peer support for physical health populations, however, the variation in population groups of included studies as well as differences in outcome measurement, make it challenging to systematize findings under one umbrella.

We also examined the impact of timing provision of peer support. O'Dell et al. reported that spinal cord injury patients were not always prepared to process information in the acute stages after injury, therefore consideration needs to be made around when the patient is the most receptive to receiving peer support [21]. No findings within included studies suggest a beneficial impact for the delivery of ‘early’ peer support on patient outcomes. This is likely to be due to the lack of focus on physical health populations in the acute stage.

Limitations

This scoping review is limited in terms of the population group considered, namely, those with physical health conditions. Alternative populations that have been shown in the literature to utilize peer support include families and carers of individuals with physical health conditions [29-31]; mental health populations [32-36]; children and young people [37-40]; ‘at-risk’ groups [41]; veterans [42] and those that are homeless [43].

## Conclusions

In conclusion, this scoping review draws attention to the difficulty in assessing the impact of peer support on general physical health populations due to the heterogeneity in participants, study design, intervention implementation, outcome measures and findings within the included studies. In terms of answering the research question, there appears to be greater consistency in the qualitative methods of the evaluation of peer support as compared to objective methods of evaluation. Synthesis of qualitative findings was grouped into general themes across included studies: (1) peer support leading to a sense of belonging or a sense of community, leading to reduced isolation and feeling understood; (2) peer support as a means of providing and/or consolidating information; (3) peer support as helpful for decision-making; (4) peer support leading to empowerment; and (5) peer support helpful for aiding self-management behaviours. The impact of peer support on objective outcome measures is mixed, with some research suggesting that peer support has a greater impact on those with higher levels of negative psychological affect than those with lower levels. Further research could look to explore this suggestion. Finally, this scoping review has highlighted the need for the piloting of peer support in acute physical health settings in order to bridge the gap that this review has identified in peer support research.

## Appendices

**Table 3:** Characteristics of sources of evidence RCT: randomized controlled trial; PS: peer support; QT: quantitative methods; QL: qualitative methods; MM: mixed methods

ID	Title	Author(s)	Country & population group	Number of study participants	Intervention	Length of time of peer support	Design and evaluation methods	Main outcomes	Main conclusions
[8]	Facilitated peer support in breast cancer: a pre- and post-program evaluation of women's expectations and experiences of a facilitated peer support program.	Power and Hegarty, 2010	Republic of Ireland, Women with primary breast cancer.	8	Format: Facilitated face-to-face peer support programme involving informational components and opportunities for sharing experiences, led by health professionals and a peer supporter who received training. 7-week PS programme led by a breast cancer nurse counsellor, a volunteer retired counsellor, a trainee counsellor and a Reach to Recovery volunteer (a breast cancer survivor who received training in providing peer support to individuals from the Irish Cancer Society). Participants met once weekly over the 7-week period for a 2.5-hour session. The programme involved a combination of information sessions on practical issues. Informal sharing of experiences between participants was encouraged by the lead facilitator.	7-weeks	QL Focus group interviews - Interviews were recorded & transcribed verbatim. Analysis: Content analysis.	Themes identified: (1) The need for mutual identification (2) Post-treatment isolation (3) Help with moving on (4) The impact of hair loss (5) Consolidation of information (6) Enablement/empowerment (7) The importance of the cancer survivor (8) Mutual sharing	Positive impact of PS: Mutual sharing of experiences, identification and understanding. Also allowed women to make definite decisions regarding important issues in their own lives. The informational component was highly valued by all and played an important role in diagnosis consolidation. Other: The need for support in relation to treatment-induced hair loss was identified. All participants would have welcomed more time to engage in ‘‘informal chatting’’ with each other.
[9]	Can peer support be cost saving? An economic evaluation of RAPSID: a randomized controlled trial of peer support in diabetes compared to usual care alone in East of England communities.	Wingate, Graffy, Holman et al., 2017	United Kingdom, People with Type 2 Diabetes	1,299 (130 clusters)	Format: Mixed delivery including 1:1 and group peer support. Peer supporters were trained and supported with meetings with a diabetes nurse. PS delivered via 1:1, group, or both group and 1:1 versus a control group receiving standard diabetes care. Intervention delivered over 8-12 months by trained PS facilitators, supported by monthly meetings with a diabetes nurse. 3 essential elements of diabetes management delivered in the first 6 months: (1) overcoming practical obstacles encountered while dealing with diabetes, (2) coping with the social and emotional aspects of diabetes and (3) the type of medical therapy used in caring for diabetes.	8-12 months	QT Economic evaluation of a 2 x 2 factorial randomised cluster-controlled trial. Out-of-pocket expenses/service utilization were self-reported at three time intervals: (1) baseline, (2) mid-point and (3) on trial completion. Non-hospital costs used NHS reference costs. Hospital payments were obtained from one local commissioning group and mean payments calculated.	(1) Out-of-pocket costs for participants: -Medications -Glucose monitoring -Costs for medical visits -Travel to appointments -Additional expenses -Total excluding covered services -Total including all costs (2) NHS incurred costs: -Accident and emergency visits -Overnight hospital stay -Nurse and GP costs -Other health professionals -Total NHS incurred costs	Positive impact of PS: decrease in systolic blood pressure (though not statistically significant) and lower overall total healthcare costs (largely due to decreased hospitalization expenses)
[10]	Effects of telephone-based peer support in patients with type 2 diabetes mellitus receiving integrated care: a randomized clinical trial.	Chan, Sui, Oldenburg et al., 2014	China, Hong Kong Chinese patients with Type 2 diabetes mellitus	628	Format: Telephone-based peer support programme. Peer supporters were trained. The programme was carried out in three diabetes centres - provided fortnightly structured comprehensive assessments via the "JADE portal" (web-based multi-component quality improvement programme). Telephone-based PS programme, PEARL (PS, Empowerment and Remote Communication linked by information technology) 33 received 32 hours of training (four 8-hour workshops) to become peer supporters (10 participants assigned to each). Peer supporters called their peers a minimum of 12 times, informed by a checklist.	12 months	QT patients randomised to either JADE & PEARL (n=312) or JADE only (n=316). All were assessed at 0 and 12 months. Analysis: Intention-to-treat analysis. The Pearson χ2 test, Mann-Whitney test, Fisher exact t-test, Wilcoxon paired test	Primary outcomes: (1) Haemoglobin level (2) Blood pressure (3) Low-density lipoprotein cholesterol ______________________________ Secondary outcomes: (1) QoL - 5-term Euro-QoL (EQ-5D) (2) Depression - Patient Health Questionnaire for depression (PHQ-9) (3) Distress - 21-item Depression Anxiety Stress Scale for Psychological Distress (DASS-21) & Chinese 15-item diabetes Distress Scale (CDDS-15) (4) Self-efficacy - 20-item Diabetes Empowerment Scale for self-efficacy (DES-20)	Positive impact of PS: Patients in both groups showed an improvement in most psychological-behavioural constructs at 12 months including medication adherence and self-efficacy. Patients with negative emotions benefited from additional PS with greater treatment compliance, improved psychological health, and reduced hospitalizations. No change: PS did not improve cardiometabolic and psychological well-being in patients with T2DM receiving integrated care.
[11]	Peer support for patients with type 2 diabetes: cluster randomised controlled trial.	Smith, Paul, Kelly et al., 2011	Republic of Ireland, Individuals with type 2 diabetes	395 (192 in the intervention group, 203 in the control group & 29 peer supporters)	Format: Peer-led face-to-face peer support programme, peer supporters were trained. Peer supporters trained on basics of Type 2 diabetes and practicalities of working in a group. PS meetings were held in General Practitioner practices. (9 PS sessions over 2 years). Meetings were led by the peer supporter (no health professionals present in the meeting room). Each session had a theme; the contents of the meeting were recorded.	2 years	QT Analysis: Multilevel linear or logistic regression models with random effects. Analysis of secondary outcomes was an intention to treat cluster-level analysis apart from analysis on BMI which was entered into the multilevel model analysis.	Primary outcomes: (1) Glycated haemoglobin (HbA1c) - measured with reverses phase cation exchange liquid chromatography with an automatic glycol-haemoglobin analyser. (2) Systolic blood pressure - measured with an automatic BP monitor. (3) Cholesterol - analysed with automated clinical chemistry analysers. (4) Wellbeing (the study doesn't report on the measure used) _______________________________ Secondary outcomes: (1) Body mass index (BMI) (2) Diabetes self-care activities (3) Self-efficacy, adherence to medications (4) Family and friends subscale of the chronic illness resources survey (5) Smoking (self-reported) (6) Prescriptions (aspirin, antihypertensive drugs, and cholesterol-lowering agents) (7) Measures of the process of care (visits to a general practitioner, practice nurse, hospital diabetes outpatients department, and hospital diabetes centre and admissions to hospital).	No changes: PS did not significantly improve physical and psychosocial outcomes or secondary outcomes. Negative impact of PS: A non-significant reduction in wellbeing in the intervention group, was shown
[12]	Puentes hacia una mejor vida (Bridges to a Better Life): Outcome of a Diabetes Control Peer Support Intervention.	Ayala, Ibarra, Cherrington et al., 2015	United States of America, Individuals with uncontrolled diabetes	336	Format: Peer-delivered peer support including telephone contact, in-person, individual and group support. Peer supporters were trained. Volunteer peer leaders worked with 5 to 8 patients each over a 12-month period (goal to achieve 8 contacts during the first 6 months). The intervention incorporated the four key functions of PS: (1) assistance with diabetes management in daily living such as problem-solving barriers to medication use; (2) social/ emotional support including how to communicate effectively with family members about one’s needs; (3) linkages to health care such as knowing where to go to obtain speciality services; (4) as well as ongoing support over time.	12 months	QT PS vs usual care. Analysis: Intention-to-treat outcome analysis.	Primary outcome: (1) Glycated haemoglobin level (HbA1c) ________________________________ Secondary outcomes: (1) Health care utilization (2) Enactment of diabetes self-management behaviours	Positive impact of PS: significant reduction in glycated haemoglobin among intervention versus usual care participants. Other: Usual care participants reported checking their feet significantly more than intervention participants.
[13]	Can a community-based peer-led diabetic self-management programme be effective: 12-week evaluation.	Shen, Wang and Edwards, 2017	China, Individuals with diabetes	181, (89 in the experimental group, 92 in the control group)	Format: Peer-delivered face-to-face group sessions with trained peer supporters. 8 peer groups, each with 12-14 peers and 2 peer leaders. During the study period, the 8 groups merged into 4 larger peer groups. Each group comprised 21-27 peers and was led by 3 or 4 peer leaders.	12 weeks	MM Analysis: ANOVA/ANCOVA and content analysis.	(1) Self-efficacy - the Chinese Diabetes self-efficacy scale (C-DSES) (2) Social support - the Medical Outcome Study Social Support Survey (MOS-SSS) (3) Self-management behaviours - the Chinese Diabetes Self-care Scale (C-DSCS) (4) QoL - The Medical Outcome Study 36-item Short-Form Health Survey (SF-36) (5) Participant's perceptions towards the programme - self-developed questionnaire consisting of choice questions and open-ended questions.	Positive impact of PS: Social support, self‐efficacy and self‐management behaviours significantly improved. Positive feedback was provided by participants. No change: Quality of life.
[14]	Telephone peer-delivered intervention for diabetes motivation and support: the telecare exploratory RCT.	Dale, Caramlau, Sturt et al., 2009	United Kingdom, Individuals with type 2 diabetes	231	Format: Telephone peer-delivered support service. Peer supporters were trained. Peers attended a two-day training programme. The course focused on the following: empowerment, motivational interviewing, and active listening skills. Books on diabetes and behaviour change were provided. In the intervention group, telecare calls were made 3-5 days later and at various time points after. The frequency of calls was tailored to patients. Record sheets were kept to monitor call content, goal setting and achievement and the length of calls.	6 months	MM Routine care versus routine care and motivational telephone support from a peer supporter or a diabetes specialist nurse (DSN) (9 peers & 12 DSNs) for a period of up to 6 months. 35 in the peer supporter group and 17 in the DSN group were invited to participate in semi-structured interviews (21 were conducted, 14 peer supporters, 7 DSNs). Interviews were also conducted with supporters 1 year into the intervention (7 peer supporters, 3 DSNs). Analysis: Linear mixed effect models for repeated measures and x2-tests. The qualitative analysis utilized a thematic framework approach.	Primary outcome: (1) Self-efficacy - the Diabetes Management Self-Efficacy Scale (DMSES) _______________________________ Secondary outcomes: (1)HbA1c (2) Patient and telecare supporter satisfaction - assessed using a non-validated questionnaire	No change: At 6 months, no significant differences in self-efficacy scores or secondary outcome measures. Other: Peer telecare support was less highly valued than support delivered by a DSN. Some patients said that they would have valued more information and advice.
[15]	Integration and Utilization of Peer Leaders for Diabetes Self-Management Support: Results from Project SEED (Support, Education, and Evaluation in Diabetes).	Rodgers, Xue et al., 2011	United States of America, People with diabetes	221	Format: Facilitated face-to-face peer support classes with peer leaders assisting educators on programme delivery. Diabetes Self-Management Education (DSME) was offered to both the intervention and enhanced usual care (EUC) groups. (1) In the intervention group (Diabetes self-management support group, DSMS), both peers and peer leaders attended four weekly DSME classes whereby peer leaders assisted the diabetes educators with class activities. (The content of the DSME and activities in the EUC group were matched to the intervention group. The difference being that the enhanced usual care group did not have peer leaders participate in any aspect). DSMS - peers attended a series of 6 monthly support group meetings. In the intervention group, peer leaders facilitated DSMS, whereas, in the enhanced usual care group, the diabetes educator facilitated sessions. (3) Telephone DSMS, following in-person support meetings, calls delivered once a month for an additional 6 months. Peer leaders facilitated all calls in the intervention group and diabetes educators made the calls in the enhanced usual care group.	12 months	QT Cluster RCT. Participants were randomized to either diabetes self-management education (DSME) & Peer leader-led diabetes self-management support (DSMS) or to enhanced usual care (n=102) Data were collected at baseline, after DSME (6 weeks), after DSMS (6 months) and after telephonic DSMS (12 months). The statistical analysis incorporated descriptive and inferential statistics.	(1) A1C (average blood glucose) - (blood samples) (2) Self-monitoring of blood glucose (SMBG) (3) Diabetes Distress - Diabetes Distress Scale (DDS)	Positive impact of PS: Peer leader-led diabetes self-management support was more effective at improving diabetes-related distress and equally effective as traditional DSME in helping participants to maintain glycaemic control and self-monitoring of blood glucose.
[16]	Perspectives of Women Considering Bilateral Prophylactic Mastectomy and their Peers towards a Telephone-Based Peer Support Intervention.	St-Pierre, Bouchard, Gauthier et al., 2018	Canada Women who had undergone or who were considering bilateral prophylactic mastectomy (PM) (cancer-free, undergone lumpectomy or partial mastectomy for breast cancer, those with a new breast cancer diagnosis who were considering bilateral PM).	19 peers, 15 recipients	Format: telephone-based peer support programme with peer supporters who had been trained Telephone-based PS intervention between women contemplating PM (recipients) and women who had undergone this surgery (peers). Telephone calls began with introductions which included surgical history and recipients were able to ask questions. Recipients also decided on the order of conversation topics and were not limited in the number of calls they could request. The study coordinator contacted each participant the day after each call to follow up about the intervention, and determine whether the recipient wanted to speak to the same peer or another peer. Peers could contact the study coordinator at any time to discuss potential issues about their supportive relationship with the recipient.	Data collected over 34 months (2.8 years)	QL Recipients questionnaire: included open-ended and closed-ended questions. Response formats for the closed-ended questions included dichotomous options and Likert scales. This questionnaire measured the number of phone calls made with the peer, topics discussed, satisfaction with the intervention, opinions about the use of the telephone, aspects of the intervention they found useful and not useful, views on the relevance of the intervention for women considering prophylactic mastectomy, and suggestions for improvement. Peer questionnaire: included open-ended and closed-ended questions. Response formats for the closed-ended questions included dichotomous options and Likert scales and similar questions to the recipient’s evaluation questionnaire. Analysis: Phenomenological. Inductive coding.	(1) Peers' Perspectives (descriptive evaluation of evaluation questionnaire) (2) Recipient's perspectives (descriptive evaluation of evaluation questionnaire)	Positive impact of PS: Recipients found the telephone-based intervention useful in their consideration of prophylactic mastectomy. PS described to “break the sense of isolation”, especially for those who did not know anybody who had undergone surgery. PS allowed recipients to feel understood by someone and helped to decrease their anxiety.
[17]	Subjective evaluation of a peer support program by women with breast cancer: A qualitative study.	Ono, Tsuyumu, Ota et al., 2017	Japan, Women with breast cancer	10	Format: Peer-delivered face-to-face peer support programme with trained peer supporters. The coordinator chose peer supporters based on the requests of patients. PS meetings lasted one approximately hour. Feedback from peers was collected by the coordinator and the details of the meeting are reported by the peer supporter. Breast cancer peer supporters attended a training course over three days. Their suitability as peer supporter was assessed in interviews by medical professionals.	13 months	QL Semi-structured interviews (lasted between 34 mins and 64 mins). Content as follows: reasons for participating in programme, content discussed in a meeting with a peer supporter, how the participant felt after receiving PS, to identify things that were good as well as things that were bad about PS and whether any improvements could be made to the programme. Analysis: Qualitative inductive analysis	Data were categorised into: (1) Benefits of the PS programme (2) Benefits of the PS programme that was received (3) Disadvantages of the PS programme	Positive impact of PS: Patients described finding PS helpful due to it being tailored to their needs as well as the programme being excellently coordinated. PS led to one “re-examining” themself and enabled one to prepare for moving forwards. Negative impact of PS: Strict management of personal information and limitations in matching.
[18]	When peer support may be most beneficial: the relationship between upward comparison and perceived threat.	Legg, Occhipinti, Ferguson et al., 2011	Australia, Breast cancer patients	251	Format: Peer-delivered face-to-face peer support with trained peer supporters. The Breast Cancer Support Service (BCSS) volunteers were breast cancer survivors who finished treatments a minimum of 12 months prior to intervention and underwent a selection and training process. Volunteers were required to reattend a minimum of 6-hour refresher training per year.	7 months	QT Perceived cancer threat was measured by The Constructed Meaning Scale – (CMS). Positive upward comparison and psychological adjustment were measured by two questions with a Likert-scale response. Analysis: Moderate multiple regression analysis.	(1) Psychological affect - The Hospital and Anxiety Depression Scale (HADS)	Positive impact of PS: Perceived cancer threat moderated the relationship between PS (positive upward comparison) and depressive levels, with those who perceived their diagnosis as more threatening and who engaged in PS reporting lower depressive levels. No change: For women who perceived their diagnosis as less threatening, PS had no impact on depression or anxiety levels.
[19]	Impact of a peer-delivered telephone intervention for women experiencing a breast cancer recurrence.	Gotay, Moinpour, Unger et al., 2007	United States of America, Women with the first recurrence of breast cancer	305	Format: Peer-delivered telephone peer support. The intervention consisted of four to eight telephone calls delivered over a one-month period. The calls were conducted by trained peer counsellors at a breast cancer advocacy organization (the Y-ME National Breast Cancer Organization) and followed a standard curriculum.	1 month	QT Outcomes were assessed at baseline, 3 and 6 months. (The 3-point assessment was the primary endpoint used in the paper). Analysis: univariate analyses.	(1) Psychological distress - Cancer Rehabilitation Evaluation System-Short Form (CARES-SF) (2) Depressive symptoms - Centre for Epidemiologic Studies Depression Scale (CES-D)	No change: No differences in distress or depressive symptoms at 3 months between the intervention and control groups.
[20]	Qualitative evaluation of a community peer support service for people with spinal cord injury.	Haas, Price and Freeman, 2013	United Kingdom, Individuals with spinal cord injuries	14	Format: No peer support programme/intervention delivered as part of the study. A qualitative evaluation of a peer support service already delivered. No specific details of the PS programme itself other than it being a PS service provided to individuals in general hospitals, who were not admitted to specialist spinal injury rehabilitation centres.	No intervention	QL Individual semi-structured in-depth interviews. Analysis: Inductive coding was used to derive themes.	Data were categorised into: (1) People with SCIs' experience of the Community PS Service (2) Relatives' experience of the Community PS Service (3) Health professionals' views of the Community PS Service	Positive impact of PS: PS was highly valued by the participants. The PS officer’s lived experience (living with a spinal injury) provided credibility to the practical advice, information and signposting and the empathy shown by them. Participants described PS as essential for others undergoing rehabilitation in hospitals.
[21]	Role of peer support for people with a spinal cord injury.	O’Dell, Earle, Rixon et al., 2019	United Kingdom, (1) People with a spinal cord injury (2) Their family and friends (3) PS officers (4) Nurse specialists (5) Other health professionals	100	Format: No peer support programme/intervention was included in this study. An evaluation of the Spinal Injury Association peer support service, using focus groups & telephone interviews. Details of the PS are not given in this paper, only that it is a PS service provided by the Spinal Injuries Association in the 10 administrative areas across England and Wales.	No intervention	QL (1) Online Survey - 48 questions, related to participants views of whether their needs were met by the PS service and as their levels of knowledge, isolation, optimism and motivation before and after contact with a PS officer. (2) A focus group, one 2-hr meeting, (3) Telephone interviews -questions for healthcare practitioners focused on the respondents’ views of what support PS officers offered to people with a spinal cord injury and how they believed PS officers supported healthcare practitioners’ roles. Questions for people with a spinal cord injury, and their family and friends related to the timing, frequency and termination of any support, and detailed examples of the nature and limitations of the support they had experienced. Analysis: Thematic analysis by constant comparisons.	Themes identified: (1) Value of shared experience (2) Providing knowledge of spinal cord injury and reducing isolation (3) Timing of support and being 'ready to talk'	Positive impact of PS: The healthcare practitioners valued the specialist training in how to support people with a spinal cord injury provided by PS officers, especially for staff in non-specialist settings. Other: Some spinal cord injury patients and healthcare practitioners agreed that patients may not always be ready to receive and process information about their injury, particularly in the acute stages.
[22]	Development and evaluation of a hospital-based peer support group for younger individuals with stroke.	Muller, Toth-Cohen and Mulcahey, 2014	United States of America, Younger individuals (<65yrs) who have had a stroke (YESS)	13	Format: Education and support group delivered by health professionals in a group setting, included a peer support module. (Not peer-led or facilitated at all). The YESS group met 9 times over an 18-week period. Each 90-minute group module focused on a specific topic. An Occupational Therapist coordinated and facilitated the group sessions which sometimes had content experts providing the primary education for specific modules. Other members of the stroke team participated and assisted in the groups.	18 weeks	MM Questionnaires (SIS and CIQ) were completed at the first and ninth group sessions. A participant feedback survey was completed once after the ninth session. Analysis: Change scores were calculated. The Wilcoxon ranked sum test was used to determine if differences in scores reached significance. The thematic analysis utilized the two open-ended questions in the survey.	(1) Healthy adjustment after stroke - The Stroke Impact Scale (SIS) (assesses 8 specific domains: hand function, strength, mobility, activities of daily living ADL, instrumental activities of daily living (iADL), memory, emotion, communication and handicap). (2) Home integration, social interaction and productivity - The Community Integration Questionnaire (CIQ) (3) Participant feedback about the process & additional evidence supporting the achievement of programme objectives, such as group members' self-perceptions of change in socialization, coping strategies and role attainment following group participation and identification of social activities outside of the group context - Survey	The positive impact of PS: Change scores of the SIS handicap domain, total the CIQ and home integration domain scores of the CIQ showed significance. A useful element from the group described was learning about new information, education, and information on community resources. Social engagement and role participation – over half reported that they began to engage in various leisure opportunities beyond the group context. No change: Change scores - The SIS self-perceived recovery score and the CIQ, social, and productivity domains did show a significant change.
[23]	Finally heard, believed and accepted--peer support in the narratives of women with fibromyalgia.	Sallinen, Kukkurainen and Peltokallio et al., 2011	Finland, Women with fibromyalgia.	20	Format: Attendees of a previous rehabilitation course with education and counselling components. Included lectures, group discussions, physiotherapy group exercises and individual treatments, participants encouraged to share experiences and continue discussions with group members. Study participants attended rehabilitation courses in Rheumatism Foundation Hospital which included PS opportunities (as well as lectures, group discussions, physiotherapy group exercises and individual treatments). Each course of 10-12 patients was completed in 17-20 days, divided into two or three intensive in-patient periods.	17-20 days	QL Narrative interview method used. Analysis: Thematic analysis.	Themes identified: (1) Permission to talk (2) Need for experiential knowledge (3) Reciprocity (4) Self-evaluation through comparison	Positive impact of PS: some participants viewed PS as a “significant turning point in their lives: they were finally heard, believed and accepted”. PS allowed participants to dare to be themselves, provided a sense of community and enhanced empowerment through validation of experiences. The negative impact of PS: Seeing others with more severe functional disabilities or depression led to the following for some; anxiety related to the future, occasional hopelessness and despair and fear of mental health problems.
[24]	Investigating the beneficial experiences of online peer support for those affected by alopecia: an interpretative phenomenological analysis using online interviews.	Iliffe and Thompson, 2019	United Kingdom, 12 recruited from the Alopecia UK Facebook Support group. 11 diagnosed with alopecia, one family member of someone diagnosed with alopecia	24	Format: No peer support programme/intervention in this study. Evaluation of the support offered by the Alopecia UK Facebook group.	No intervention	QL Semi-structured interviews through Facebook messenger. The interview schedule consisted of open-ended questions and prompts. Analysis: Interpretative Phenomenological Analysis	Themes identified: (1) Gradual healing (2) Image concern (3) Belonging (4) New identity and self-acceptance	Positive impact of PS: created a feeling of belonging, which could lead to a sense of acceptance and feeling.
[25]	Nurse-led peer support group: experiences of women with polycystic ovary syndrome.	Percy, Gibbs, Potter et al., 2009	United Kingdom, Female patients with polycystic ovary syndrome (POCS)	13	Format: Facilitated face-to-face peer-support programme led by nurses with a heavy focus on providing information and components of peer support. PS groups were monthly and were open to females with POCS and their friends/family members. Meetings usually started with an invited speaker e.g. dietician or endocrinologist, question and answer session and unstructured group discussion.	2 months - 2 years	QL Semi-structured interview design. Questions covered multiple topics including related to: current experience of POCS; expectations and aspirations for POCS; experiences of POCS services and suggestions for improving POCS services. Analysis: Inductive and deductive qualitative analysis.	Themes identified: (1) Expectations and hopes (2) Socioemotional function (3) Informational function (4) Personal impact of the support group (5) Criticisms and suggestions for change	Positive impact of PS: PS helped to reduce isolation, and provided an opportunity for social comparison and accessible and personally relevant information. PS had a major personal impact for some participants. Some reported feeling empowered as well as having direct positive effects on their self-management behaviours.
[26]	Qualitative insights into implementation, processes, and outcomes of a randomized trial on peer support and HIV care engagement in Rakai, Uganda.	Monroe, Nakigozi, Ddaaki et al., 2017	Uganda, People living with HIV who have not yet initiated antiretroviral therapy (ART).	75, 41 in-depth interviews with 39 participants including people living with HIV (n=23), peer supporters (n=9) and staff (n=7).	Format: Qualitative evaluation following RCT with quantitative trial findings. No peer support intervention in this study. The intervention (first part of the exploratory study) consisted of monthly structured home visits by peers to intervention arm participants to provide psychosocial support and promote engagement in HIV care (e.g. attending clinic appointments) and a basic care package of preventive care items including cotrimoxazole prophylaxis, safe water vessel use, insecticide-treated bed net use, and condoms.	No intervention	QL Part of a wider study that utilized a MM design (the first part is reported elsewhere). (1) In-depth interviews (2) Group discussions around specific topics from the in-depth interviews.	Themes identified: (1) Information (2) Motivation (3) Behavioural skills (4) Situated factors (2) Challenges and areas for improvement (3) Trial insights	Positive impact of PS: PS improved information, motivation, and behavioural skills, leading to increased engagement in pre-ART care. Some participants reported that peer supporters helped to reinforce health messages and enabled them to better understand complicated health information as well as helping participants to navigate the health system, develop support networks, and identify strategies for remembering medication and clinic appointments. PS improved client engagement in care, cotrimoxazole use, and safe water vessel use. Other: Practical challenges of PS delivery were found: insufficient messaging surrounding ART initiation, lack of care continuity after ART initiation, rare breaches in confidentiality, and structural challenges.
[27]	Peer support to promote physical activity after completion of centre-based cardiac rehabilitation: evaluation of access and effects.	Clark, Munday and McLaughlin et al., 2012	United Kingdom, People with heart disease who had completed centre-based cardiac rehabilitation	109	Format: Facilitator-led face-to-face peer support programme with group-based health education delivered by health professionals & support from peer mentors who had been trained. Patients were matched with peers who were former patients who had completed centre-based cardiac rehabilitation 1 to 2 years previously and were still participating in community-based physical activity at local municipally funded gymnasia or other fitness facilities throughout the region. Peer mentors engaged in an afternoon training programme provided by health professionals of the centre-based cardiac rehabilitation team who explained mentor roles and responsibilities and highlighted key physical activity research-based principles and messages.	12 months	QT Longitudinal pre-test post-test design, with measurements of physical activity at baseline and after 12 months. For one week, participants were asked to wear a pedometer and maintain a physical activity log for seven consecutive days at baseline and follow up. Statistical analysis: Paired t-tests from baseline to 12 months, two-sample t-tests for comparisons of Programme versus non-programme users, Chi-squared analysis and Pearson correlations to analyse for relationships between pedometer and physical activity data.	(1) Physical activity level - 7-day Physical Activity Recall Questionnaire & pedometers. (2) Support for physical activity - The Social Support in Exercise Survey	Positive impact of PS: PS participants provided more total physical activity versus the non-programme group (though did not reach statistical significance). No change: No difference between groups in total amounts of work-related physical activity or leisure-related activity at 12 months. Social support levels were also similar between groups at 12 months and no change in either group from baseline levels. Negative impact of PS: At 12 months, pedometer count and average step count remained higher in the non-programme group versus the programme group. Other: Women were significantly more likely to join the PS program compared to men. A significant decrease in physical activity levels was shown in the non-programme group.
[28]	Long-Term Social Reintegration Outcomes for Burn Survivors with and Without Peer Support Attendance: A Life Impact Burn Recovery Evaluation (LIBRE) Study.	Grieve, Shapiro, Wibbenmeyer et al., 2020	United States of America, Burn survivors	601	Format: No peer support/programme. Cross-sectional study that evaluated "peer support attendance" with no peer support attendance on societal reintegration. Collected data for participation on PS, no direct PS programme/intervention detailed.	No intervention	QT Statistical analysis: chi-square tests and multivariable linear regression models.	(1) Social participation - The Life Impact Burn Recovery Evaluation Profile	Positive impact of PS: Burn survivors who reported attendance to PS had higher social interaction scores than those who did not. Attendees reported fewer restrictions in participating in social activities, relating and maintaining friendships, and dealing with strangers compared with burn survivors who reported no peer group exposure.
